# Hybrid Alginate–Graphene Composites: Biochemical Features and Biomedical Potential

**DOI:** 10.3390/md23080323

**Published:** 2025-08-09

**Authors:** Marcin H. Kudzin, Anna Kaczmarek, Zdzisława Mrozińska, Cesar Hernandez, Klaudia Piekarska, Katarzyna Woźniak, Michał Juszczak, Paulina Król

**Affiliations:** 1 Łukasiewicz Research Network, Lodz Institute of Technology, 19/27 Marii Sklodowskiej-Curie Str., 90-570 Lodz, Poland; 2 Department of Molecular Genetics, Faculty of Biology and Environmental Protection, University of Lodz, 90-236 Lodz, Poland

**Keywords:** activated partial thromboplastin time, alginic acid, blood coagulation, calcium, cell viability, composite material, DNA damage, Hs68 cells, PBM cells, polymer functionalisation, prothrombin time

## Abstract

Alginate-based materials are widely studied for biomedical use, but their limited mechanical properties and variable biocompatibility pose challenges. In this work, hybrid composites composed of alginate, calcium, and graphene oxide were fabricated using a freeze-drying method and cross-linked with calcium ions via calcium chloride at different concentrations. Structural and morphological features were assessed using SEM, EDS, ICP-MS, and BET analysis. The resulting composites exhibited a porous architecture, with calcium incorporation confirmed by elemental analysis. Surface characteristics and pore parameters were influenced by the presence of graphene oxide and the cross-linking process. The effects of the materials on haemostasis were evaluated through activated partial thromboplastin time (aPTT) and prothrombin time (PT) assays, revealing modulation of the intrinsic coagulation pathway without significant changes in the extrinsic pathway. In this study, we analysed the effect of alginate–graphene oxide composites on the viability of peripheral blood mononuclear (PBM) cells and human foreskin fibroblasts from the Hs68 cell line. We also assessed the genotoxic potential of alginate–graphene oxide composites on these cells. Our results showed no cyto- or genotoxic effects of the material on either cell type. These findings suggest the biocompatibility and safe character of alginate–graphene oxide composites for use with blood and skin cells.

## 1. Introduction

**Figure 1:**
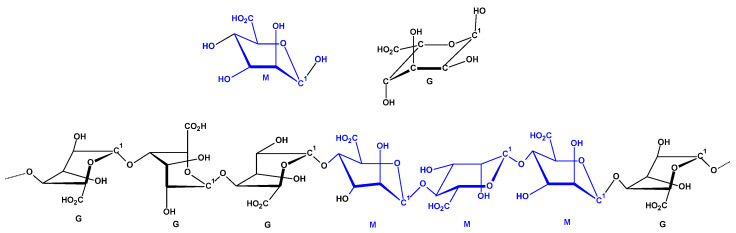
Structures of alginate epimers: α-L-guluronic acid residues (G, GulA) and β-D-mannuronic (M; ManA) and alginic acid -G-G-G-M-M-M-G- fragments drawn by means of chair conformation (C-H bonds are omitted).

Excessive bleeding due to traumatic injuries is a leading cause of pre-hospital fatalities globally [1]. Consequently, it is crucial to implement effective haemostatic strategies to reduce the mortality associated with significant blood loss [2,3,4,5,6]. Among the various materials employed as haemostatic agents, natural polymers are frequently favoured due to their biocompatibility, biodegradability, versatility in fabrication, and their capacity to activate the coagulation cascade [7,8]. Among these is alginate, which is the second most abundant biopolymer, composed of blocks of β-D-mannuronate (M) and α-L-guluronate (G) residues linked together [9,10,11,12,13] (see Figure 1).

Chemical adaptability, biocompatibility, biodegradability, non-immunogenicity, non-toxicity, and biological activity of alginates recommended this carbohydrates for drug delivery (5294 document results on alginate according to Scopus base; 386 since 2021) [14]; wound dressing delivery (1392 document results on alginate for wound dressings according to Scopus base, 127 in 2021) [15], or tissue engineering (3749 document results on alginate for tissue engineering according to Scopus base; 291 in 2021) [16].

**Figure 2:**
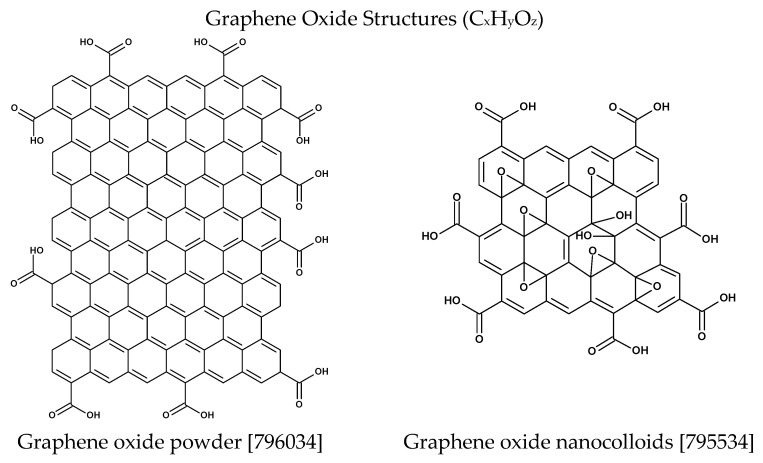
Structure of graphene oxide materials manufactured by Merck Group [27].

Graphene oxide (GO) is an oxidised derivative of graphene with a two-dimensional sheet structure composed of a single layer of carbon atoms with oxygen-containing functional groups [17,18,19] (Figure 2). It is a highly versatile and tunable material with unique physical and chemical properties [20,21,22,23], which has attracted considerable attention in the field of biomaterials [20,24,25,26].

Due to the presence of numerous hydrophilic end-groups, such as hydroxyl, epoxy, and carboxyl groups, GO has high water affinity [28,29]. This, combined with the hydrophobic backbone, makes GO amphiphilic [28]. High specific surface area, layered structure, along with the presence of the oxygen-containing functional groups, enabling covalent, electrostatic, and hydrogen bonding, make GO an excellent absorbent of different biomolecules such as proteins, nucleic acids, as well as polysaccharides [30,31].

It has been demonstrated that polymeric scaffolds modified with graphene oxide exhibit a huge potential in terms of tissue engineering [30,32]. Additionally, the superior toughness and tensile properties of graphene oxide substantially improve the mechanical strength of polysaccharide materials [33,34]. Moreover, some studies highlight a high potential of GO for haemostatic applications. High water affinity provides GO with very good liquid absorbability [29]. As a result, graphene oxide has the capability to quickly absorb plasma and concentrate blood cells, platelets, coagulation factors, and various other mass components on its surface, thereby facilitating the coagulation process [28,29]. The potential of GO-based materials as wound dressings, as a result of rapid absorption of liquids, was confirmed in other reports [29,35]. Moreover, graphene oxide is capable of activating platelets and initiating the coagulation cascade [28,29,36]. As explained by Singh [37], it facilitates platelet activation and aggregation by stimulating Src kinase and promoting the release of intracellular calcium. Furthermore, it was demonstrated that it can induce a similar effect as thrombin upon interaction with blood. The study carried out by Zhang et al. [38] indicated that an increased quantity of GO resulted in higher platelet activation and enhanced coagulation efficiency of a composite sponge made from N-alkylated chitosan and graphene oxide [38]. Moreover, it was reported that GO may improve the antibacterial properties of a polymer matrix [39].

Therefore, the combination of alginates and graphene oxide resulted in many biomedical applications of these composites, which are represented by examples given in Table 1.

The main aim of this study was to develop alginate–GO composites cross-linked with calcium ions using the freeze-drying technique and to assess their biological and biochemical properties. This study was focused on the impact of the alginate–GO on the blood coagulation parameters and the influence of post-incubation mixtures of alginate–GO on cell viability and DNA damage in PBM cells and skin fibroblasts from the Hs68 cell line as a model of normal human cells. The production of post-incubation mixtures was selected as the most appropriate way to evaluate the effect of alginate–GO composites on human cells. Moreover, the physicochemical properties of the developed foams were investigated, including chemical composition, surface morphology, specific surface area, and porosity parameters.

## 2. Results and Discussion

### 2.1. Preparation of Graphene Oxide–Calcium Alginate Composites and Their Structural Characterisation

**Figure 3:**
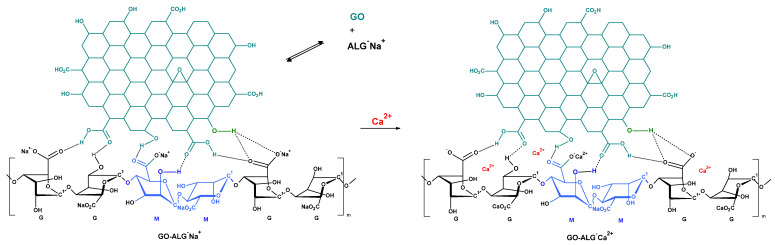
Schematic illustration of the preparation of structure of the graphene oxide–calcium alginate composites (for other graphene oxide–alginate hybrid structural projections see papers [40,41,51,52,53]). Dotted lines represent hydrogen bonds.

Graphene oxide–calcium alginate composites were prepared in the following reaction sentence: GO → GO-ALG^−^ Na^+^ → GO-ALG^−^ Ca^2+^ → GO-ALG^−^ Ca^2+^ _(gel)_ (Figure 3). The applied reaction conditions are summarised in Table 2 (concentration of cross-linking calcium ions, reaction temperature, and time (for detailed data see Section 3)).

The physico-optical evaluation of the obtained samples revealed the following optimal parameters of the cross-linking reaction: 40 °C, 60 s reaction time, and a calcium chloride concentration of 0.5% to 2.0%.

### 2.2. Chemical and Structural Characterisation

#### 2.2.1. Measurement of Calcium Concentration

The results on calcium content in GO-ALG-Ca composites obtained in these reaction conditions are listed in Table 3.

As can be observed in Table 3, the calcium content in the samples increases with the rise in the concentration of the CaCl_2_, i.e., the cross-linking agent. The lowest concentration of calcium, i.e., 8562 mg/kg, was obtained in the case of the ALG_GO_0 sample, which was not subjected to cross-linking with Ca^2+^. Samples cross-linked with calcium ions were characterised by a significantly higher Ca content, i.e., 51,820 mg/kg (ALG_GO_C, 0.5% CaCl_2_), 75,278 mg/kg (ALG_GO_B, 1% CaCl_2_), and 93,776 mg/kg (ALG_GO_A, 2% CaCl_2_). The presented results are in agreement with the findings obtained for sodium alginate foams without a bulk modifier, i.e., graphene oxide, cross-linked with Ca^2+^ using CaCl_2_ [54].

#### 2.2.2. Morphology and Elemental Analysis

**Figure 4:**
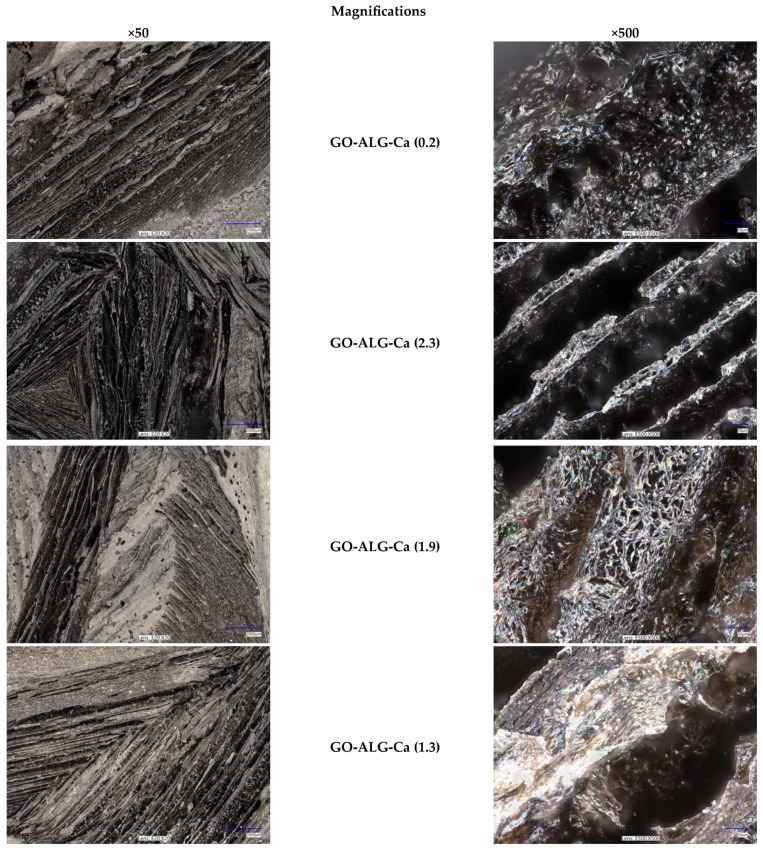
Optical microscopy images (magnifications: ×50, ×500) of samples before and after the modification processes.

The morphology of the sodium alginate foams obtained by means of the freeze-drying technique was observed using the optical digital microscope (Figure 4).

The black colouring of the samples is due to the applied bulk modifier, i.e., graphene oxide flakes. For all of the foams, both unmodified and cross-linked with calcium ions, the typical sponge-like, porous structure was observed. According to the literature, the foam is defined as a composite structure consisting of gas-filled voids (dispersed phase) embedded within a denser, continuous medium, which is commonly either a liquid or a solid [55,56]. As can be observed in Figure 4, the developed alginate–GO composites are characterised by a porous structure with interconnected pores; this confirms their cellular nature, which is typical for foam materials. Therefore, it can be concluded that the developed alginate–GO composites meet the definition of a foam, as they are built from an alginate matrix with clearly visible pores (i.e., voids filled with air).

Under low magnification, the characteristic longitudinal fissures were visible, creating thin lamellae. This structure is due to the freeze-drying process, during which ice crystals are formed within the sodium alginate and then removed in order to obtain the desired porous structure [54]. Despite the difference in the concentration of the CaCl_2_ in the cross-linking solution, there was no distinctive difference in the structure of the samples.

**Figure 5:**
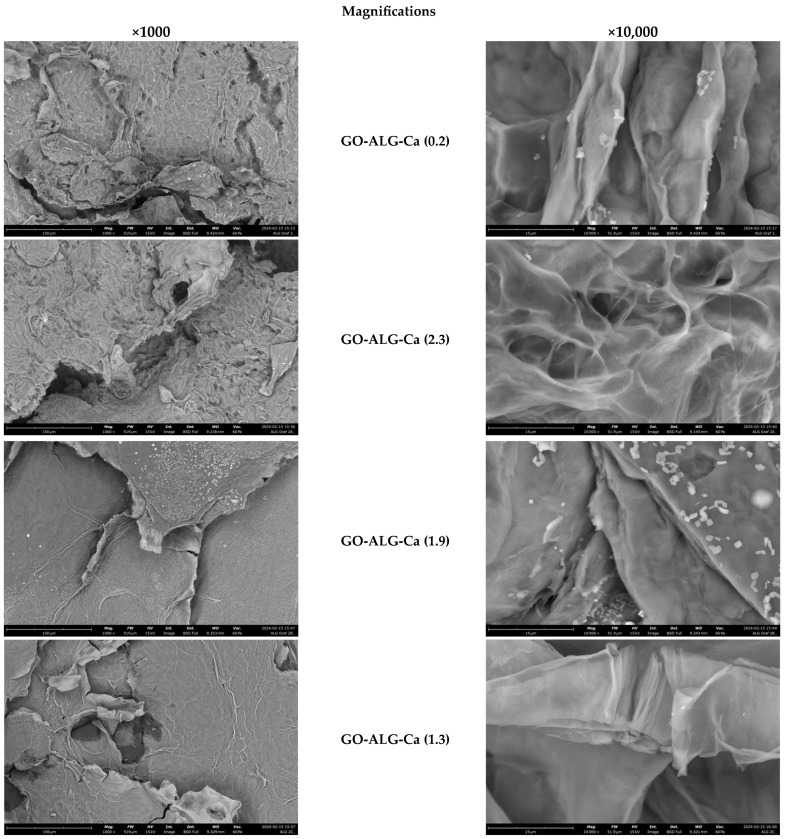
SEM images (magnifications: ×1000, ×10,000) of samples before and after the modification processes.

Detailed analysis of the surface morphology and structure of the manufactured composites was possible by means of scanning electron microscopy (Figure 5). It was revealed that all the samples are characterised by an irregular, corrugated surface. The surfaces of both unmodified and modified composites were rough and porous with clearly visible cracks and voids. Moreover, some agglomerates were visible. Considering the presence of the agglomerates on the surface of all the examined foams, it may be assumed that the agglomerates were graphene oxide flakes, which were added to the polymer solution to obtain sodium alginate–GO composites.

The obtained results differ from the findings presented in our previous work, in which sodium alginate foams (without the addition of graphene oxide as a bulk modifier) were cross-linked with Ca^2+^ using a CaCl_2_ solution [57]. In the case of the sodium alginate foams without the GO modifier, it was observed that cross-linking with calcium ions resulted in the formation of a calcium ion network within the alginate structure, which in turn caused significant surface modifications. It was observed that the porosity of the samples increases with the increasing concentration of the CaCl_2_. This was associated with increased cross-link density and higher material stiffness and rigidity.

However, in the case of the sodium alginate foams with the addition of a bulk modifier, i.e., GO flakes, there was no distinctive difference in the surface morphology that could be attributed to the formation of the calcium ion network. This may be explained by the high addition of the bulk modifier, i.e., 24.5 wt. %, which may have masked the influence of the cross-linking process. As a result, the surface morphology and structure of the sodium alginate–GO foams were dependent on the composition of the sodium alginate solution and the freezing-drying process itself.

**Figure 6:**
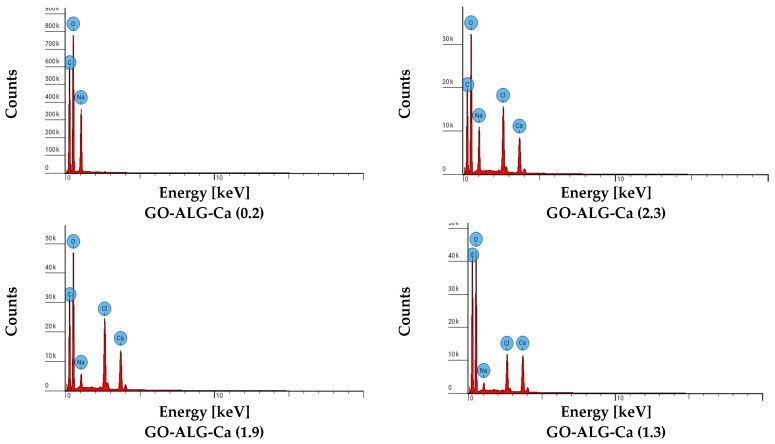
EDS spot analysis diagrams of GO-ALG-Ca/Na composites.

The qualitative analysis of the chemical composition of the composites was performed using energy-dispersive X-ray spectroscopy (EDS). The obtained spectra are illustrated in Figure 6. EDS spot analysis data of GO-ALG-Ca/Na composites are presented in Table 4. For all of the samples, the peaks corresponding to oxygen, carbon, and sodium were observed. All those elements are constituents of sodium alginate and graphene oxide, which are the main components of the composite foams. For the samples cross-linked with Ca^2+^ (using CaCl_2_), additional peaks attributed to calcium and chloride appeared. This confirms that the CaCl_2_ was successfully incorporated within the composites’ structure, and the cross-linking of the sodium alginate–graphene oxide composites with calcium and chloride ions occurred. Moreover, the relative height of the peaks originating from Ca and Cl increases with the increasing concentration of CaCl_2_ in the cross-linking solution. At the same time, it can be observed that the relative height of the peak corresponding to sodium decreases, which may be associated with the substitution of sodium in the sodium alginate structure by Ca and Cl.

**Figure 7:**
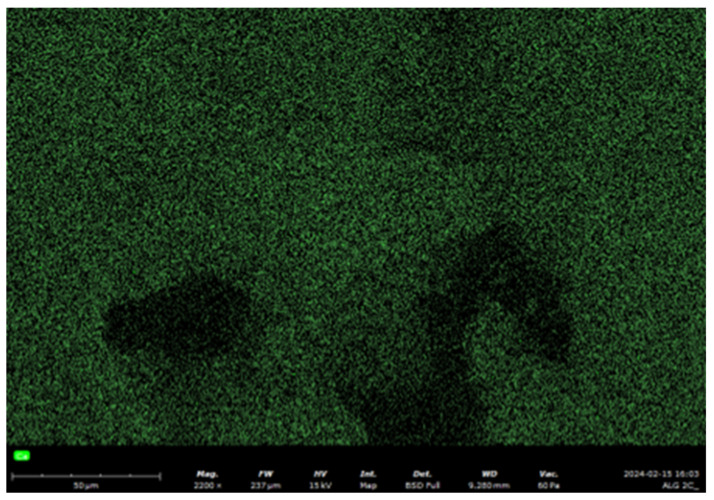
SEM-EDS elemental mapping of calcium (green) for the GO-ALG-Ca (2.3) composite. Magnification: 2200.

In addition to spot analysis, we also performed elemental mapping using EDS coupled with SEM to visualise the distribution of calcium ions in selected samples. Figure 7 presents a representative EDS elemental map of calcium (green signal) in the GO-ALG-Ca (2.3) composite. The calcium signal is evenly distributed across the surface, confirming homogeneous incorporation of calcium ions into the polymer matrix during cross-linking. This result is consistent with the EDS point measurements (Table 3), which show increasing Ca content with higher CaCl_2_ concentrations, and supports the effectiveness of the cross-linking procedure at the structural level. The mapping also confirms the absence of localised Ca-rich agglomerates, suggesting a uniform ion exchange process between sodium alginate and calcium ions throughout the matrix.

#### 2.2.3. Chemical Structure Analysis (FTIR)

**Figure 8:**
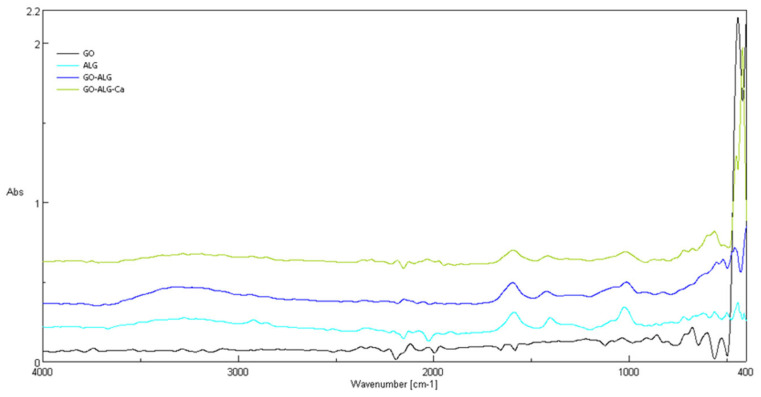
FTIR spectra of the GO and GO–ALG–Na samples and the GO-ALG-Ca and GO-ALG-Ca composites.

To confirm the chemical structure and interactions within the composite materials, Fourier-transform infrared spectroscopy (FTIR) was conducted for sodium alginate (ALG), graphene oxide (GO), the alginate–GO composite (GO-ALG), and the cross-linked composite with calcium chloride (GO-ALG-Ca (2.3)) (Figure 8).

Spectra were collected over the 4000–400 cm^−1^ range, and the main features are summarised below. The FTIR spectrum of sodium alginate shows a broad O–H stretching band around 3200–3400 cm^−1^ and two intense carboxylate bands at approximately 1590 cm^−1^ and 1400–1420 cm^−1^. These features, typical for alginates [9,11,31,58], correspond to asymmetric and symmetric stretching vibrations of –COO^−^ groups and associated C–OH deformation. A distinct peak at about 1030 cm^−1^ arises from C–O stretching within the polysaccharide backbone [59].

Graphene oxide exhibits weaker shoulders near 1720 cm^−1^ and 1615 cm^−1^—bands associated with carbonyl C=O and aromatic C=C stretching [58]. More pronounced absorptions at roughly 1220 cm^−1^ and 1040–1050 cm^−1^ are attributable to C–O–H and epoxy C–O groups [58,60]. These features confirm the presence of various oxygen-containing functional groups in oxidised graphene. When alginate and GO are combined, the resulting composite spectrum incorporates elements from both materials. Carboxylate bands appear at approximately 1596 cm^−1^ and 1423 cm^−1^, and the C–O stretching region (1010–1080 cm^−1^) remains prominent. Broadening of the C–OH band near 1200 cm^−1^ suggests hydrogen-bonding interactions between alginate chains and GO sheets, in agreement with earlier studies on alginate/GO composites [31]. A broad O–H band is also visible around 3300 cm^−1^ [60]. Calcium cross-linking brings further spectral changes. In GO-ALG-Ca, the carboxylate bands shift slightly to about 1594 and 1417 cm^−1^ and become broader, consistent with ionic coordination of Ca^2+^ to carboxylate groups [11,61]. A shoulder in the 1430–1450 cm^−1^ region and additional low-frequency bands below 500 cm^−1^ reflect these Ca–O interactions [61]. Collectively, these shifts and broadenings confirm successful incorporation of GO into the alginate matrix and formation of Ca^2+^-mediated cross-links.

#### 2.2.4. Specific Surface Area and Total Pore Volume Analysis

**Figure 9:**
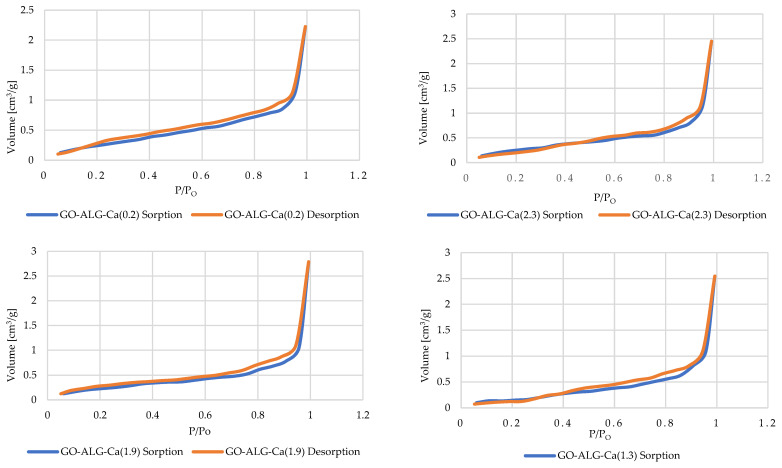
Sorption and desorption isotherms of samples before and after the modification processes.

To assess the porosity of the developed sodium alginate–GO foams, the Brunauer–Emmett–Teller (BET) analysis was conducted. This allowed us to evaluate not only the impact of the cross-linking process on the specific surface area of the composites, but also the total pore volume and the average pore diameter. The obtained results are presented in Table 5, while Figure 9 presents the N_2_ adsorption–desorption isotherms of the examined samples.

As far as the specific surface area is concerned, the lowest concentration of the CaCl_2_ in the cross-linking solution resulted in the initial decrease in the specific surface area from 1.29 to 0.94 m^2^/g (for GO-ALG-Ca (0.2) and GO-ALG-Ca (1.3), respectively). For samples modified using higher CaCl_2_ concentration, the specific surface area increased slightly to 1.03 and 1.02 m^2^/g (for GO-ALG-Ca (1.9) and GO-ALG-Ca (2.3), respectively). At the same time, it is worth noting that according to our previous results, the mean specific surface area for the sodium alginate foam without the addition of the GO modifier was significantly lower, i.e., equal to 0.33 m^2^/g [49]. This implies that the use of a bulk modifier influences the specific surface area of the sodium alginate foams developed using the freeze-drying technique. The increase in the specific surface area may be associated with the appearance of the GO agglomerates on the surface. Moreover, our earlier results revealed that the surface of the sodium alginate foam without a modifier is relatively smooth and uniform [57], while the surface of the sodium alginate–GO foam was characterised by a more irregular surface with considerable wrinkling, which also affects the specific surface area. Similarly, the total pore volume of the sodium alginate–GO foams (3.45 × 10^−3^ cm^3^/g) is notably higher than for the unmodified sodium alginate foams (1.31 × 10^−3^ cm^3^/g [57]). Furthermore, the cross-linking process resulted in the further increase in the total pore volume up to 3.95 × 10^−3^, 4.32 × 10^−3^ cm^3^/g, and 3.80 × 10^−2^ cm^3^/g for GO-ALG-Ca (1.3), GO-ALG-Ca (1.9), and GO-ALG-Ca (2.3) foams, respectively. This is in accordance with our previous results, which demonstrated that higher CaCl_2_ concentration led to a higher cross-link density followed by a bigger porosity associated with the formation of the calcium ion network [57]. The observed increase in the total pore volume may also be associated with the increase in the average pore diameter (from 12.6 nm for the GO-ALG-Ca (0.2) sample to 21.6, 20.1, and 15.2 nm for the GO-ALG-Ca (1.3), GO-ALG-Ca (1.9), and GO-ALG-Ca (2.3) samples, respectively).

The N_2_ adsorption–desorption isotherms obtained for the unmodified and modified sodium alginate–GO foams are presented in Figure 9. Similarly, as in the case of the sodium alginate foams [57], all of the observed isotherms may be categorised as type III isotherms according to the International Union of Pure and Applied Chemistry (IUPAC) classification [55,56]. Type III isotherms are characterised by the hyperbolic graph with no distinct “knee” formed, and are typical for non-porous and/or macroporous materials. The occurrence of this type of isotherm is usually associated with relatively weak interactions between the adsorbent and adsorbate [55,56]. Based on the shape of the isotherms, it may be observed that for lower pressures, a slow increase in the quantity of adsorbate occurred, while for higher pressures, a sharp, exponential increase in the sorption appeared. The absence of a so-called “knee” may be attributed to the multilayer sorption mechanism [55,56]. Furthermore, for all the examined samples, the presence of a hysteresis loop was observed. This is due to the capillary condensation effect [55,56,62]. The observed hysteresis loops were identified as the type H3 hysteresis loop, which is associated with the slit-shaped pores and indicates that the macrospores were not fully filled with condensate.

The relatively low specific surface area values obtained in our BET measurements are most likely due to the freeze-drying method and the high alginate concentration used during gelation, both of which lead to dense polymeric matrices with limited accessible internal surface. This effect has been documented in previous studies of alginate scaffolds prepared by lyophilisation, showing that lower biopolymer concentrations tend to yield higher porosity and surface area [63,64].

### 2.3. Biological and Biochemical Properties

#### 2.3.1. Blood Plasma Clotting: aPTT and PT

**Figure 10:**
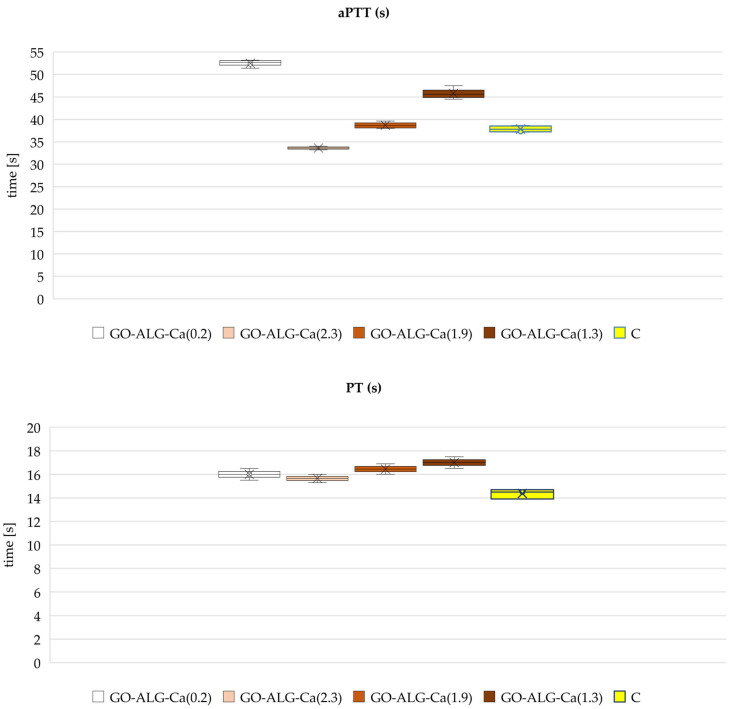
Effect of ALG_GO composites on activated partial thromboplastin time (aPTT) and prothrombin time (PT). The results are presented as mean (×), median (horizontal line), range (bars), and interquartile range (box).

Evaluating the hemocompatibility of the developed alginate materials is critical for their prospective medical applications, such as wound dressings. To assess their impact on blood coagulation and potential thrombogenicity, in vitro coagulation assays were performed. The assays included measuring activated partial thromboplastin time (aPTT) and prothrombin time (PT). Activated partial thromboplastin time (aPTT) is a widely used laboratory test that evaluates the functionality of the intrinsic coagulation pathways [65,66,67]. This assay measures the time required for plasma to clot after the addition of an activator (such as kaolin or silica), phospholipid, and calcium ions [65,66,67]. The intrinsic pathway involves coagulation factors XII, XI, IX, and VIII. Prothrombin time (PT) assesses the extrinsic coagulation by measuring the clotting time after the addition of tissue factor (thromboplastin) to the plasma. The extrinsic pathway primarily involves factor VII. By measuring both aPTT and PT, it is possible to evaluate the influence of the alginate materials on multiple coagulation pathways, thus determining their safety profile concerning blood compatibility and thrombogenic risk. The obtained results are presented in Figure 10.

As it may be observed, the shortest aPTT was measured for the composite made of alginate and graphene oxide (GO-ALG-Ca (0.2)). According to the literature, alginate–GO composites have demonstrated rapid haemostatic effects in vitro, effectively stopping bleeding within seconds [68]. This rapid haemostasis is particularly beneficial in the case of wound dressings, especially in terms of emergency care and surgical applications. The presence of graphene oxide in the composite is believed to enhance platelet aggregation, which promotes clotting [35,36,38,69,70,71,72]. It is believed that graphene oxide supports coagulation mainly by facilitating platelet function. Singh et al. [37] indicated that GO can trigger a significant aggregatory response in platelets, which may be linked to the release of intracellular free calcium from cytosolic stores and the activation of non-receptor protein tyrosine kinases belonging to the Src family in platelets. At the same time, the impact of alginate–GO composites on prothrombin time (PT) is minimal, indicating that the extrinsic coagulation pathway is not significantly affected. This may be due to the fact that alginate alone, as well as combined with GO, does not interfere with clotting factors involved in PT.

When calcium ions are present in the composite, such as in calcium chloride (CaCl_2_), they act as cofactors that accelerate clotting via the intrinsic pathway, leading to a shortened aPTT [57]. This is due to the fact that calcium ions are essential cofactors in blood coagulation [32,73,74]. Such results have been previously reported by the authors for the Ca^2+^ cross-linked alginate composites obtained via the freeze-drying technique. It has been revealed that aPTT decreases with higher concentrations of calcium ions, i.e., CaCl_2_ [57]. However, in the case of the alginate–GO composites cross-linked with calcium ion, it was observed that the aPTT initially prolongs. This may be explained by the fact that alginate, being a negatively charged polysaccharide, can tightly bind calcium ions [75,76,77]. This binding could reduce the availability of free calcium needed to activate coagulation factors, prolonging aPTT despite the presence of Ca^2+^. Additionally, functional groups of graphene oxide may interact with calcium [78,79], further modulating calcium ion availability. As a result, we suppose that the composite matrix might slow the release or availability of calcium ions into the plasma, causing delayed or insufficient activation of the intrinsic pathway, which can lead to prolonged aPTT. However, with the increasing concentration of CaCl_2_ in the cross-linking solution, the aPTT shortens, which may be associated with the higher availability of calcium ions, which is consistent with the previously obtained results [57]. Unlike aPTT, prothrombin time (PT) showed no significant alterations in response to different concentrations of calcium ions, i.e., CaCl_2_.

#### 2.3.2. Effect of Alginate–GO Foams on the Viability of PBM and Hs68 Cells

**Figure 11:**
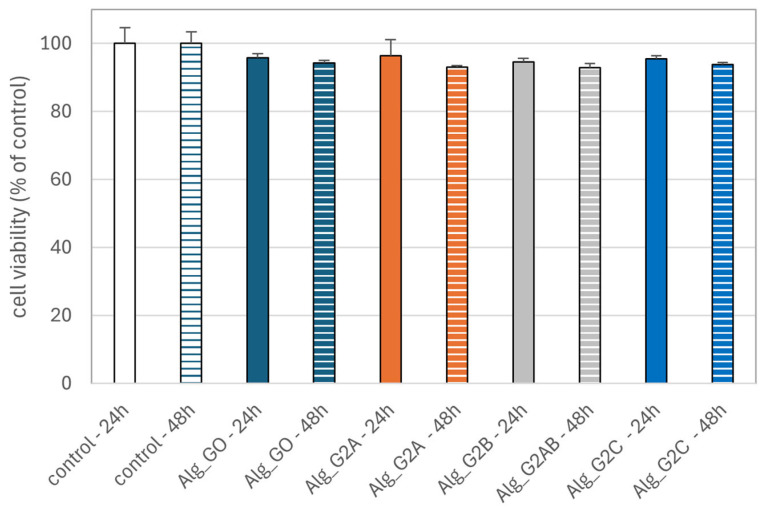
Effects of alginate–GO foam post-incubation mixtures Alg_GO (GO-ALG-Ca (0.2)), Alg_G2A (GO-ALG-Ca (2.3)), Alg_G2B (GO-ALG-Ca (1.9)), and Alg_G2C (GO-ALG-Ca (1.3)) on PBM cell viability after 24 and 48 h of incubation. The results are presented as the mean result from 6 repeats. Error bars denote SD.

**Figure 12:**
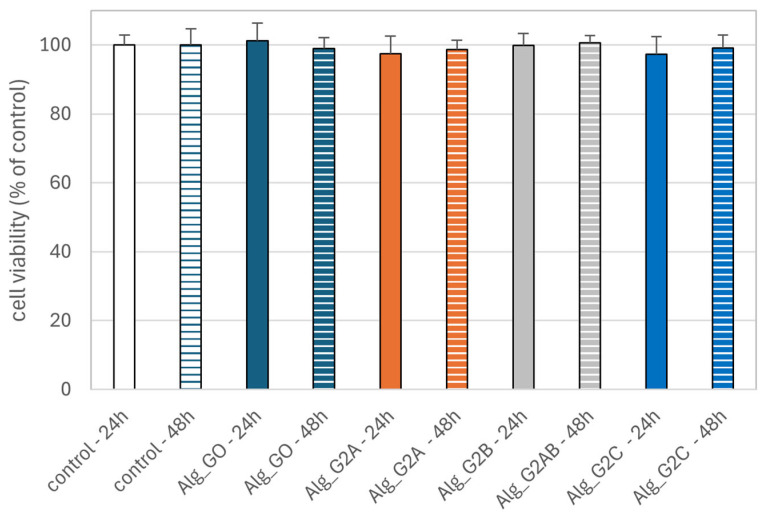
Effects of alginate–GO foam post-incubation mixtures Alg_GO (GO-ALG-Ca (0.2)), Alg_G2A (GO-ALG-Ca (2.3)), Alg_G2B (GO-ALG-Ca (1.9)), and Alg_G2C (GO-ALG-Ca (1.3)) on Hs68 cell viability after 24 and 48 h of incubation. The results are presented as the mean result from 6 repeats. Error bars denote SD.

We used the resazurin reduction assay to determine cell viability after incubation with alginate–GO post-incubation mixtures. This assay is based on the application of an indicator dye to measure oxidation–reduction reactions, which principally occur in the mitochondria of live cells. The non-fluorescent dark blue dye (resazurin) becomes fluorescently pink at 570 nm and fluorescently red at neutral pH (resorufin) when reduced by metabolically active cells. We showed that incubation of PBM cells with alginate–GO post-incubation mixtures did not decrease cell viability after 24 and 48 h (Figure 11) of incubation. We obtained comparable results in the case of Hs68 cells (Figure 12). Our results indicate the absence of cytotoxic properties of alginate–GO materials against both types of cells. These results suggest that graphene present in the tested alginate–GO does not lead to cytotoxic effects in normal cells. This is important because data are available on the cytotoxic effects of graphene oxide against blood and skin cells [20,80,81,82].

#### 2.3.3. Effect of Alginate–GO Foams on DNA Damage in PBM Cells and Hs68 Cells

**Figure 13:**
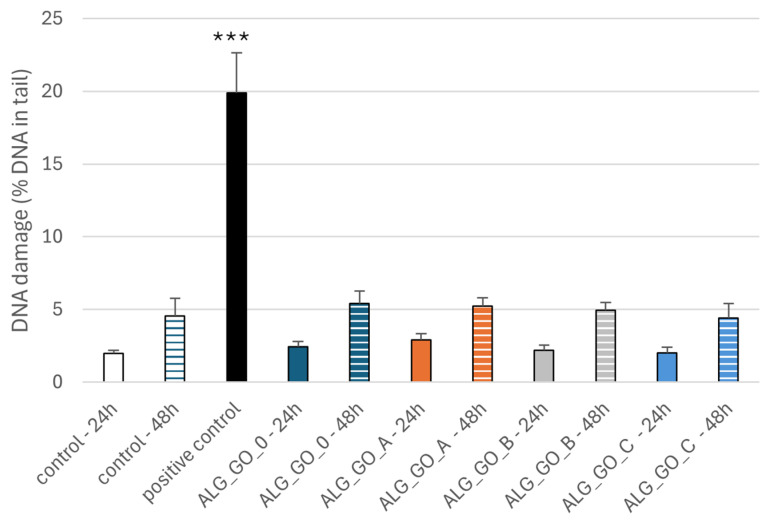
Effects of alginate–GO post-incubation mixtures Alg_GO_0 (GO-ALG-Ca (0.2)), Alg_GO_A (GO-ALG-Ca (2.3)), Alg_GO_B (GO-ALG-Ca (1.9)), and Alg_GO_C (GO-ALG-Ca (1.3)) on DNA damage in PBM cells after 24 and 48 h of incubation. The results are presented as mean results from 100 comets. Error bars denote SEM; *** *p* < 0.001.

**Figure 14:**
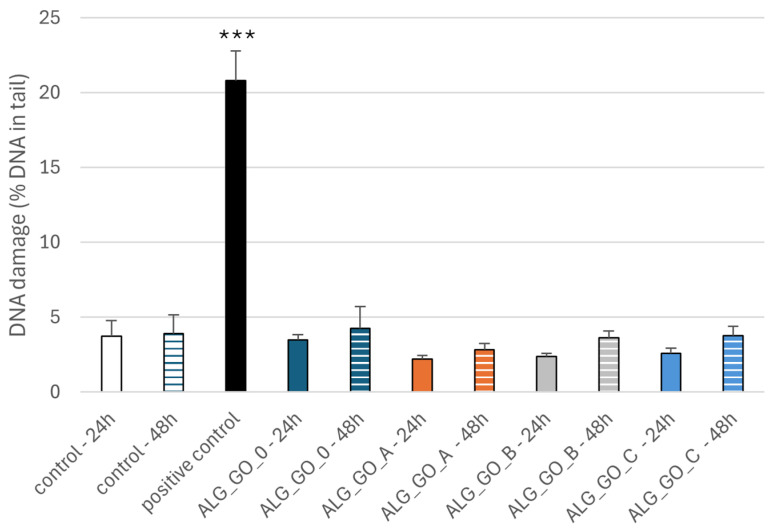
Effects of alginate–GO post-incubation mixtures Alg_GO_0 (GO-ALG-Ca (0.2)), Alg_GO_A (GO-ALG-Ca (2.3)), Alg_GO_B (GO-ALG-Ca (1.9)), and Alg_GO_C (GO-ALG-Ca (1.3)) on DNA damage in Hs68 cells after 24 and 48 h of incubation. The results are presented as mean results from 100 comets. Error bars denote SEM; *** *p* < 0.001.

**Figure 15:**
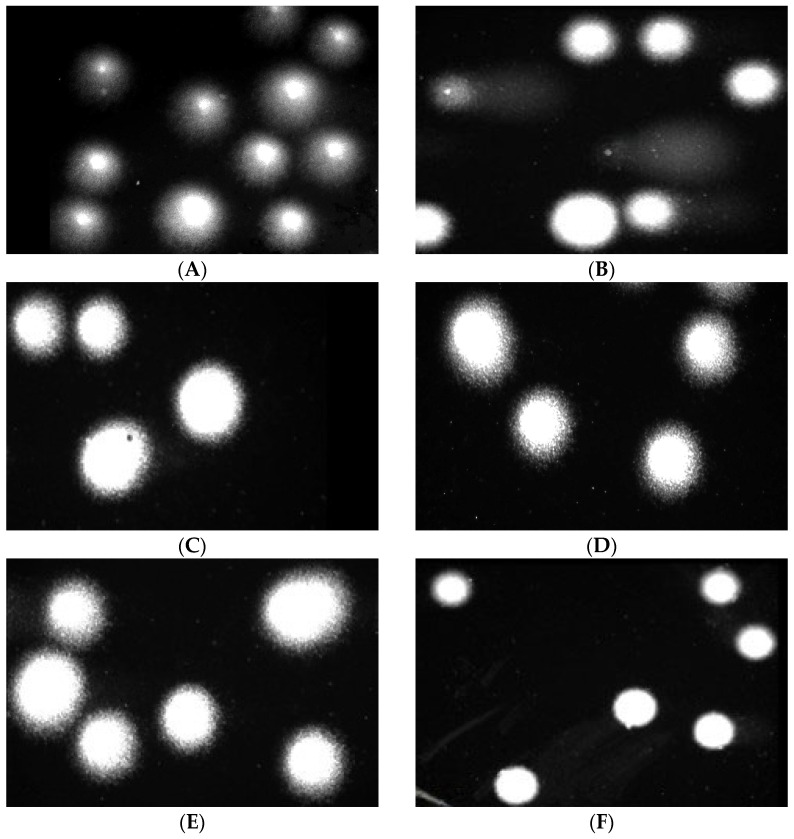
Effects of medium (**A**), 25 µM hydrogen peroxide (**B**), GO-ALG-Ca (0.2) (**C**), GO-ALG-Ca (2.3) (**D**), GO-ALG-Ca (1.9) (**E**), and GO-ALG-Ca (1.3) (**F**) post-incubation mixtures on DNA damage in PBM cells after 24 h of incubation.

**Figure 16:**
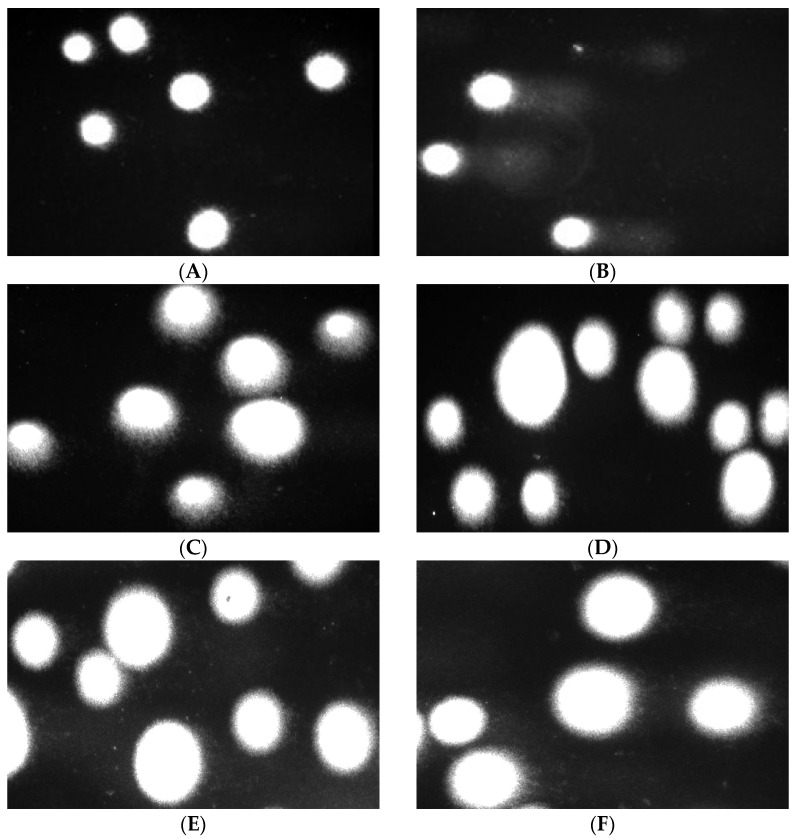
Effects of medium (**A**), 25 µM hydrogen peroxide (**B**), GO-ALG-Ca (0.2) (**C**), GO-ALG-Ca (2.3) (**D**), GO-ALG-Ca (1.9),(**E**) GO-ALG-Ca (1.3) and (**F**) post-incubation mixtures on DNA damage in Hs68 cells after 24 h of incubation.

The comet assay in the alkaline version is a sensitive and simple method of determining the level of DNA damage, including single- and double-strand breaks and alkali-labile sites in living cells [83]. We observed severe DNA damage in PBM cells and Hs68 cells incubated with 25 µM H_2_O_2_ (positive control). In the case of alginate–GO post-incubation mixtures, we did not observe an increase in DNA damage after 24 and 48 h (Figure 13 and Figure 14) of incubation in both cell lines. Moreover, we present pictures of comets (Figure 15 and Figure 16) that show no DNA damage in the case of post-incubation mixtures, whereas hydrogen peroxide-induced severe DNA damage is visible. Alginate–GO showed that the tested alginate–calcium–graphene materials did not exhibit DNA damage. Available sources indicate graphene oxide’s ability to induce DNA damage [84,85,86].

## 3. Materials and Methods

### 3.1. Preparation of the Composite Material

#### 3.1.1. Materials

**Figure 17:**
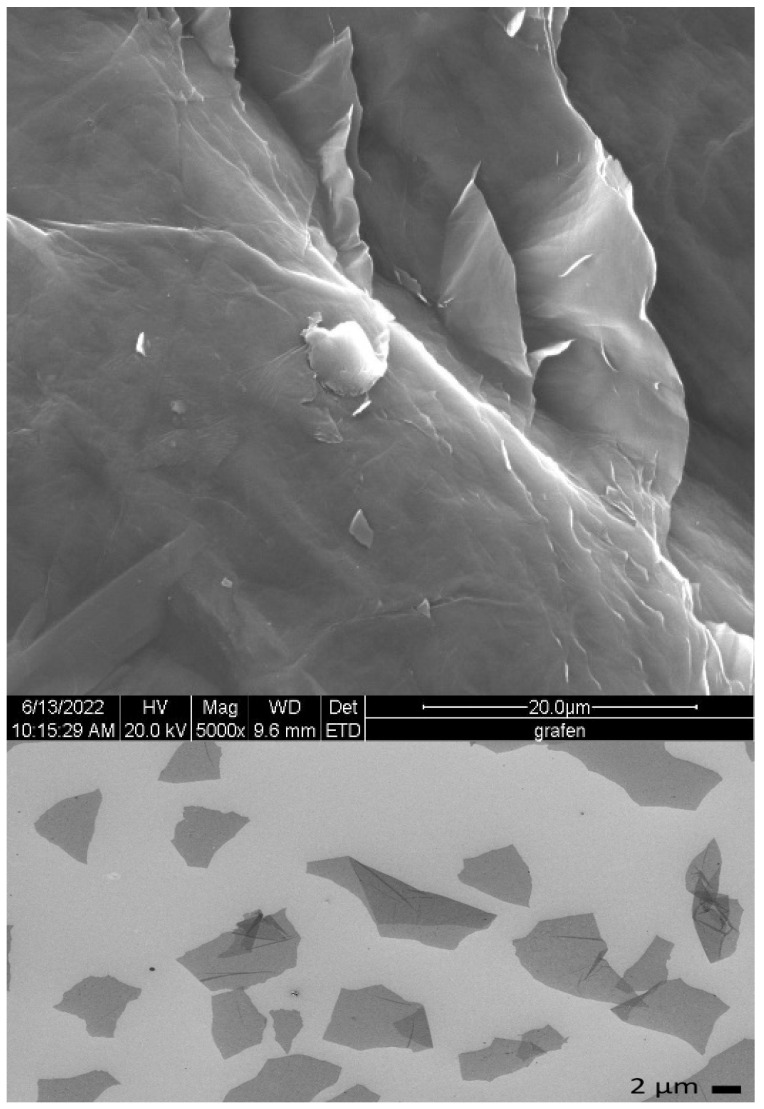
SEM image of graphene oxide (GO) flakes.

- In this study, alginic acid sodium salt from brown algae (sodium alginate) (Merck, Darmstadt, Germany) with viscosity 5.0–40.0 cps for c = 1% in water, 25 °C, was used as a base polymer to prepare porous polymer matrices (foams).
- Graphene oxide flakes in dispersion (Łukasiewicz Research Network—Institute of Microelectronics and Photonics, Graphene and Composites Research Group, presented in Figure 17) with the trade name G-Flake were added to the polymer solution in the amount of 24.5 wt. % [87,88].
- Calcium chloride anhydrous (Merck, Darmstadt, Germany) was used as a cross-linking agent with 3 different concentrations: 0.5, 1, and 2%.
- Standard human blood plasma lyophilisates (Dia-CONT I), aPTT reagent (Dia-PTT), PT reagent (Dia-PT), TT reagent (Dia-TT), 0.025 M CaCl_2_ solution reagent from Dia-PTT (Diagon Kft, Budapest, Hungary), and a coagulometer (K-3002 OPTIC, KSELMED^®^, Grudziądz, Poland) were used for the aPTT and PT measurements according to the manufacturer’s instructions.

#### 3.1.2. Solutions

- Sodium Alginate 2% wt. % Solution: Sodium alginate (2 G) was added in portions to water (98 G) during slow stirring. Afterwards, the mixture was stirred vigorously and simultaneously heated to a temperature of about 40 °C for about 4–6 h, using a mechanical stirrer.
- Sodium Alginate–Graphene Oxide Solution: GO flake dispersion solution was diluted with water and homogenised using an ultrasonic probe for up to 20 min. Then, an ALG Na solution (2% wt. %) was added in portions, stirring slowly. Afterwards, the solution was stirred vigorously and simultaneously heated to a temperature of about 40 °C for about 4–6 h, using a mechanical stirrer.
- Calcium Alginate and Graphene Oxide–Calcium Alginate: A highly porous sodium alginate foam was prepared by the freeze-drying technique [89], based on a sodium alginate polymer solution in water (2%). As modifiers, for bulk modification as well as for polymer cross-linking agents, such as G-flakes (24.5 wt. %) (Figure 17) and calcium chloride were used. GO was added to the polymer solutions and stirred vigorously for 5 h. The foams obtained by the method in [89] were then cross-linked using an aqueous solution of calcium chloride. The cross-linked foam samples were rinsed in distilled water until the chlorides were washed out, and then subjected to freezing and freeze-drying.

To diversify the calcium content in the final samples, 3 different cross-linking factor concentrations (0.5%, 1%, and 2%), 4 different coagulation durations (30 s, 60 s, 120 s, and 300 s), and temperature conditions to soften or intensify coagulation conditions (4 °C and 40 °C) were applied.

Mild coagulation conditions (lower calcium chloride concentration and lower temperature) result in a lower degree of calcium ion substitution. By increasing the temperature and calcium chloride concentration (tightening the coagulation conditions), samples with a higher calcium content are obtained. The coagulation time is also a crucial factor that determines the degree of substitution of sodium alginate with calcium ions.

Using the method in [54], a porous material is obtained in the form of foam with significant porosity up to 96%, characterised by a system of connected pores with pore sizes ranging from 0 to 359 µm.

### 3.2. Chemical and Structural Characterisation

#### 3.2.1. Inductively Coupled Plasma Mass Spectrometry Method (ICP-MS)

Calcium content in the composite samples was evaluated using a single-module Magnum II microwave mineralizer (Ertec, Wrocław, Poland). Sample digestion occurred in a closed system, which facilitated precise control over temperature and pressure. This was achieved using the Magnum II mineralizer, with the addition of 2.5 mL of 65% nitric acid (HNO_3_) and 2.5 mL of hydrogen peroxide (H_2_O_2_). The quantitative assessment of calcium (Ca) was conducted using an ICP-MS 7900 (Agilent Technologies, Santa Clara, CA, USA). All measurements were performed in duplicate, and the average values were reported as the results.

#### 3.2.2. Morphology and Elemental Analysis

The analysis of surface morphology of the tested samples was conducted using both optical and scanning electron microscopy techniques. Optical microscopy was performed with a VHX-7000N digital microscope (Keyence, Osaka, Japan) at a magnification of 50× and 500×. Scanning electron microscopy (SEM) analysis of the composites was executed using a Phenom ProX G6 scanning electron microscope (Thermo Fisher Scientific, Waltham, MA, USA). SEM imaging was conducted under low vacuum conditions (60 Pa) with a probe beam energy of 15 keV. The back-scattered electron detector was employed, and magnifications of 1000× and 10,000× were utilised. The qualitative analysis of the chemical composition was performed with the EDS X-ray microanalyzer from Oxford Instruments (Abingdon, UK).

#### 3.2.3. Chemical Structure Analysis (FTIR)

The chemical surface structure of the tested samples was assessed via ATR-FTIR using Jasco’s 4200 (Tokyo, Japan) spectrometer, with a Pike Gladi ATR attachment (Cottonwood, AZ, USA), in the range 400–4000 cm^−1^.

#### 3.2.4. Specific Surface Area and Total Pore Volume Analysis

The Brunauer–Emmett–Teller (BET) method was utilised to assess the specific surface area and total pore volume. Measurements were conducted using an Autosorb-1 instrument (Quantachrome Instruments, Boynton Beach, FL, USA), with nitrogen at 77 K acting as the adsorption agent. Before the analysis, the samples were dried at 105 °C for a duration of 24 h. Subsequently, the samples were degassed at ambient temperature. Approximately 2 g of each sample was weighed and used for the measurements.

### 3.3. Biological and Biochemical Properties

#### 3.3.1. Blood Plasma Clotting: aPTT and PT

Human plasma that had been previously frozen and freeze-dried (Dia-CONT I, Diagon Kft, Budapest, Hungary) was dissolved in 1 mL of deionised water.

To perform the experiment, 1 mL of deionised water was added to the alginate sample (weight of 6 mg), vortexed for 1 min, and then incubated for 15 min at 37 °C. Next, 100 µL of the sample was taken from the alginate solution prepared in this way and added to 100 µL of plasma. As a result, a test solution with an alginate concentration of 3 mg/mL was obtained. Then, the sample was vortexed and incubated again for 15 min at 37 °C. The control samples were treated in the same way.

The aPTT measurements were performed for each sample. First, 50 µL of alginate plasma sample and 50 µL of Dia-PTT (Diagon Kft, Budapest, Hungary) reagent were added to a cuvette located in the coagulometer thermostat (37 °C) and incubated for 3 min. The measurements were initiated by adding 50 µL of 0.025 M CaCl_2_. The PT measurements were performed by firstly incubating the alginate plasma sample (50 µL) at 37 °C in the coagulometer thermostat for 2 min, and next adding the 100 µL Dia-PT (Diagon Kft, Budapest, Hungary).

#### 3.3.2. Preparation of Composites for Assessment of Biological Properties

To analyse the influence of alginate–GO material on cells, 1 mg of fragments of alginate–GO were incubated with 1 mL of RPMI or DMEM medium, depending on the cell line, at 37 °C in 5% CO_2_ for 24 h. After that, post-incubation mixtures were filtered with a 0.2 µm filter to obtain an aseptic condition. Then, post-incubation mixtures were added to cells in a ratio of 1:9 to analyse their influence on cell viability and DNA damage.

#### 3.3.3. Cell Culture

Peripheral blood mononuclear cells (PBM cells) were isolated from a leukocyte buffy coat collected from the blood of healthy non-smoking donors from the Blood Bank in Lodz, Poland, as described previously [90]. The first step of isolation of the PBM cells was the preparation of a mixture of fresh blood from buffy coats with PBS at a ratio of 1:1. In the next step, the mixture was centrifuged in a density gradient of Lymphosep (Cytogen, Zgierz, Poland) at 2200 RPM for 20 min with the lowest values of acceleration and deceleration. Then, PBM cells were washed three times by centrifugation with 1% PBS. After isolation, the cells were suspended in RPMI 1640 medium. The University of Lodz Research Ethics Committee approved this study (12/KEBN-UŁ/I/2024–2025).

The human foreskin fibroblast line Hs68 (ATCC^®^ CRL-1635™) was obtained from the American Type Culture Collection (ATCC™, Manassas, VA, USA). Hs68 cells were cultured in DMEM high glucose medium supplemented with 100 units of potassium penicillin, 100 μg of streptomycin sulphate per 1 mL of culture media, and 10% (*v*/*v*) FBS. Cultures were maintained at 37 °C in a humidified atmosphere with 5% CO_2_.

#### 3.3.4. Cell Viability Resazurin Assay

The cell viability resazurin assay was performed using the method described by O’Brien et al. [91]. Resazurin salt powder was dissolved in a sterile PBS buffer. Post-incubation mixtures were added to PBM cells in a count of 5 × 10^4^ and to Hs68 cells in a count of 1 × 10^4^, and then incubated for 24 and 48 h at 37 °C in 5% CO_2_. The negative control was RPMI1640 for PBM cells and DMEM for Hs68 medium, which were prepared in the same manner as the post-incubation mixtures. Next, 10 µL of resazurin salt was added to each well, and the plates were again incubated at 37 °C in 5% CO_2_ for 2 h. Next, fluorescence was measured with an HT microplate reader, BioTek Synergy HT (Agilent Technologies, Inc., Santa Clara, CA, USA), using λ_ex_ = 530/25 and λ_em_ = 590/35 nm. The effects of alginate–GO post-incubation mixtures were quantified as the percentage of control fluorescence.

#### 3.3.5. DNA Damage

Alginate–GO post-incubation mixtures were added to PBM cells at a count of 7.5 × 10^4^ and to Hs68 cells in a count of 3.75 × 10^4^, and then incubated for 24 and 48 h at 37 °C in 5% CO_2_. The negative control was RPMI1640 for PBM cells and DMEM for Hs68 medium, which were prepared in the same manner as the post-incubation mixtures. The experiment included a positive control, which was a cell sample incubated with hydrogen peroxide (H_2_O_2_) at 25 μM for 15 min on ice.

The comet assay was performed under alkaline conditions [92,93]. A freshly prepared cell suspension in 0.75% LMP agarose dissolved in PBS buffer was layered onto microscope slides (Superior, Langenfeld, Germany), which were pre-coated with 0.5% NMP agarose. Then, the cells were lysed for 1 h at 4 °C in a buffer containing 2.5 M NaCl, 0.1 M EDTA, 10 mM Tris, and 1% Triton X-100, with pH = 10. After cell lysis, the slides were placed in an electrophoresis unit. DNA was allowed to unwind for 20 min in the solution containing 300 mM NaOH and 1 mM EDTA, with pH > 13. Electrophoretic separation was performed in the solution containing 30 mM NaOH and 1 mM EDTA, pH > 13, at an ambient temperature of 4 °C (the temperature of the running buffer did not exceed 12 °C) for 20 min at an electric field strength of 0.73 V/cm (28 mA). Then, the slides were washed in water, drained, stained with 2 µg/mL DAPI, and covered with coverslips. To prevent additional DNA damage, the procedure described above was conducted under limited light or in the dark.

The comets were observed at 200× magnification using an Eclipse fluorescence microscope (Nikon, Tokyo, Japan) attached to a COHU 4910 video camera (Cohu, Inc., San Diego, CA, USA) equipped with a UV-1 A filter block and connected to a personal computer-based image analysis system, Lucia-Comet v. 7.0 (Laboratory Imaging, Praha, Czech Republic). One hundred images (comets) were randomly selected from each sample, and the mean value of DNA in the comet tail was taken as an index of DNA damage (expressed in per cent).

## 4. Conclusions

The obtained results confirm that the structural properties and ionic composition of the developed GO-ALG-Ca/Na composites are strongly influenced by the composition of the cross-linking bath. Increasing the concentration of Ca^2+^ ions led to higher calcium content within the matrix, as confirmed by ICP-MS and EDS analyses. This also contributed to changes in the porosity and internal morphology of the materials, which were effectively stabilised during the freeze-drying process. We hypothesised that combining alginate with graphene oxide and applying controlled ionic cross-linking would result in porous, structurally coherent materials with enhanced potential for biological interactions. FTIR spectroscopy confirmed the presence of characteristic functional groups and the formation of ionic bonds between calcium ions and carboxyl moieties in alginate. Moreover, the presence of GO introduced additional surface functionalities, which may play a role in future biochemical or antimicrobial activity. The novelty of this work lies in the use of a straightforward and scalable freeze-drying technique to obtain ionically cross-linked alginate–graphene oxide composites with tunable architecture, without the need for supercritical processing. The ability to precisely control ion content, internal structure, and composite homogeneity offers a promising platform for the design of biofunctional materials, particularly in applications where porosity, ion exchange capacity, and surface activity are critical. Preliminary biological evaluations provided further insight into the safety and bioactivity of the developed composites. Plasma coagulation assays indicated that the materials primarily influence the intrinsic coagulation pathway, without significantly disturbing the extrinsic one—an important consideration for blood-contacting applications. Moreover, cytotoxicity and genotoxicity tests revealed no adverse effects of the graphene-containing materials on PBM and Hs68 cells. These findings confirm the biocompatibility of the composites and support further in vitro and in vivo studies to evaluate their full biomedical potential.

## Figures and Tables

**Table 1:** Biomedical applications of ALG-G and ALG-GO composites.

Composite	Biomedical Application	Lit.
GO-ALG-Ca^2+^	Bioengineering applications	[40]
rGO-ALG-Ca^2+^	Potential for cardiac patch application	[41]
GO-DFO-ALG-Mg^2+^	Bone defect regeneration	[42]
GO-ALG-Zn^2+^	Antibacterial activity and cytotoxicity	[43]
GO-Ag-ALG-Na^+^	Antibacterial activity	[44]
ALG-Na^+^-CTS-Col-GO; ALG-Ca^2+^-CTS-Col-GO	Scaffolds for bone tissue engineering	[45]
Gel/ALG/GO *	Scaffold for neural tissue repair	[46]
GO-ALG-Zn^2+^	Prevents *Staphylococcus aureus* and MRSE	[47]
G-ALG-Ca^2+^	Neural applications	[48]
G-ALG-Na^+^	Resveratrol delivery	[49]
G-HP-ALG-Na^+^	For medical applications	[50]

Col—collagen; CTS—chitosane; DEF—deferoxamine; G—graphene; Gel—gelatine; GO—graphene oxide; GO *—carboxyl graphene oxide; rGO—reduced graphene oxide; HP—hydroxyapatite; and MRSE—methicillin-resistant Staphylococcus epidermidis.

**Table 2:** Parameters of the preparation of GO-ALGCa_(gel)_.

ALGNa → GO-ALGNa→ GO-ALGNa → GO-ALGCa → GO-ALGCa_(gel)_				
ALGNa → GO-ALGNa		GO-ALGNa → GO-ALGCa → GO-ALGCa_(gel)_		
Coordination of GO by ALG		Cross-linking reaction		
ALG:GO		Ca^2+^ concentr.	Reaction temp.	Reaction time
		0.5%, 1%, and 2%	4 °C, 40 °C	30, 60, 120, and 300

**Table 3:** The results of the determination of calcium content in the alginate composite samples by ICP-MS.

Sample Symbol	Cross-Linking Agent Conc.	Calcium Content in Polymer		GO-ALG-Ca (mM *) ^/a^
	[%]	[mg/kg]	Mmol/kg (mM *)	
ALG_GO_0	no agent (0% CaCl_2_)	8.562	0.21	GO-ALG-Ca (0.2)
ALG_GO_C	0.5% CaCl_2_	51.820	1.3	GO-ALG-Ca (1.3)
ALG_GO_B	1% CaCl_2_	75.278	1.88	GO-ALG-Ca (1.9)
ALG_GO_A	2% CaCl_2_	93.776	2.34	GO-ALG-Ca (2.3)

The atomic mass of calcium (Ca) is approximately 40.078 u. mM *—milimolal concentration (mmol/kg). ^/a^ Composites GO-ALG-Ca^2+^ and/or GO-ALG-Na^+^ are presented as GO-ALG-Ca and/or GO-ALG-Na, respectively.

**Table 4:** EDS spot analysis of GO-ALG-Ca/Na composites.

Samples	Element Contents (Atomic Conc.)				
	C	O	Na	Ca	Cl
GO-ALG-Ca (0.2)	48.651	40.089	1.348	4.113	5.799
GO-ALG-Ca (1.3)	53.053	36.851	2.436	4.283	3.397
GO-ALG-Ca (1.9)	46.162	39.926	4.389	3.923	5.600
GO-ALG-Ca (2.3)	50.918	41.243	0.746	3.970	3.124
**Samples**	**Element Contents (weight Conc.)**				
	**C**	**O**	**Na**	**Ca**	**Cl**
GO-ALG-Ca (0.2)	35.908	39.418	1.906	10.130	12.638
GO-ALG-Ca (1.3)	40.200	37.200	3.600	11.400	7.600
GO-ALG-Ca (1.9)	33.601	38.716	6.118	9.529	12.036
GO-ALG-Ca (2.3)	39.239	42.342	1.101	10.210	7.107

**Table 5:** The specific surface area and total pore volume for the unmodified alginate sample and the GO-ALG-Ca/Na composites.

Sample Name	Specific Surface Area (SSA)	Total Pore Volume (TPV)	Average Pore Diameter (APD)
	m^2^/g	cm^3^/g	nm
GO-ALG-Ca (0.2)	1.2920	3.453 × 10^−3^	12.6
GO-ALG-Ca (2.3)	1.0222	3.798 × 10^−2^	15.2
GO-ALG-Ca (1.9)	1.0332	4.322 × 10^−3^	20.1
GO-ALG-Ca (1.3)	0.9401	3.948 × 10^−3^	21.6

The results were measured in duplicate and are presented as a mean value with ± deviation equal to approximately 2%.

## Data Availability

The original contributions presented in this study are included in the article. Further inquiries can be directed to the corresponding author.
